# Predictors of Poststroke Health-Related Quality of Life in Nigerian Stroke Survivors: A 1-Year Follow-Up Study

**DOI:** 10.1155/2014/350281

**Published:** 2014-05-28

**Authors:** Ashiru Mohammad Hamza, Nabilla Al-Sadat, Siew Yim Loh, Nowrozy Kamar Jahan

**Affiliations:** ^1^Center for Population Health, Department of Social and Preventive Medicine, Faculty of Medicine, University of Malaya, 50603 Kuala Lumpur, Malaysia; ^2^Department of Rehabilitation Medicine, Faculty of Medicine Building, University of Malaya, 50603 Kuala Lumpur, Malaysia; ^3^SEACO/School of Medicine and Health Sciences, Monash University Malaysia, 146 Jalan Sia Her Yam, Segamat District, 85000 Jahor State, Malaysia

## Abstract

This study aims to identify the predictors in the different aspects of the health-related quality of life (HRQoL) and to measure the changes of functional status over time in a cohort of Nigerian stroke survivors. A prospective observational study was conducted in three hospitals of Kano state of Nigeria where stroke survivors receive rehabilitation. The linguistic-validated Hausa versions of the stroke impact scale 3.0, modified Rankin scale, Barthel index and Beck depression inventory scales were used. Paired samples *t*-test was used to calculate the amount of changes that occur over time and the forward stepwise linear regression model was used to identify the predictors. A total of 233 stroke survivors were surveyed at 6 months, and 93% (217/233) were followed at 1 year after stroke. Functional disabilities were significantly reduced during the recovery phase. Motor impairment, disability, and level of depression were independent predictors of HRQoL in the multivariate regression analysis. The involvement of family members as caregivers is the key factor for those survivors with improved functional status. Thus, to enhance the quality of poststroke life, it is proposed that a holistic stroke rehabilitation service and an active involvement of family members are established at every possible level.

## 1. Introduction

Stroke-related mortality and morbidity in late adulthood continue to be a public health problem in many developing countries. Increasing prevalence of modifiable risk factors such as hypertension, obesity, and physical inactivity has contributed to the global burden of stroke which becomes one of the top three leading causes of adult death. Stroke along with other chronic diseases like cardiovascular, cancer, and diabetes will continue to augment the burden of chronic diseases in low and middle income countries [1–3]. Today, stroke has become one of the major contributors of disease burden of African countries [4–6]. Its prevalence has also started to increase in Nigeria, with prevalence of 1.14 per 1000 while a 30-day case fatality rate is as high as 40% [7]. Several hospital-based researches have found that stroke is one of the major neurological causes of admission and case fatality in Nigeria currently [8–11]. Nevertheless, due to improved treatment and management, stroke-related mortality is declining, leaving many survivors disabled [12, 13].

The impact of stroke on different dimensions of health-related quality of life (HRQoL) of survivors varies during the recovery phase when an individual attempts to adjust to life. Hence, HRQoL has become one of the key indicators to measure the poststroke outcome. Assessment of HRQoL is multidimensional, focusing on physical, mental, functional, and social aspects. It is important to know how different dimensions of HRQoL vary over time among survivors. Even though, evidence on the consequences of stroke and its determinants on HRQoL among long term survivors had already been generated in developed country; research has been sparse in developing countries [14, 15].

Studies on stroke in Nigeria have included the estimation of the stroke prevalence in urban and rural settings [16–18] and the identification of the profile and determinants of health-related quality of life (HRQoL) among stroke survivors [19, 20]. So far to our knowledge, no prospective longitudinal research has been conducted to measure the changes that occur within one year period in the different aspects of health-related quality of life among the Nigerian stroke survivors. Therefore, this study is aimed to measure specifically the changes that occur on different aspects of HRQoL and to identify the factors that predict those changes on their physical, mental, and social health during the recovery phase. The study findings are expected to generate evidence in support of developing effective health policy and strategy related to rehabilitation facilities and services for the stroke survivors who need support to maintain their optimal physical, mental, and social functional levels.

## 2. Methodology

A prospective longitudinal observational study was conducted in the department of Medicine and Physiotherapy of one teaching and two specialist hospitals of Kano state, one of the most populated states of northwestern Nigeria. These three hospitals treat and manage most of the referred stroke patients and provide rehabilitation service. Ethical approval was obtained from the Medical Ethics Committee of the University of Malaya, Malaysia (Ethics Committee/IRB Ref. number 830.7) and also from the Ministry of Health of Kano State, Nigeria (HMB/GEN/488/11) and Aminu Kano Teaching Hospital of Nigeria (AKTH/MAC/SUB/12A/P3/IV/801).

## 3. Study Population and Recruitment

Study respondents were patients with the diagnosis of stroke admitted into the hospitals between November 2010 and January 2011. Inclusion criteria are first-ever stroke patients, who were at the age of 18 years and above and were receiving rehabilitation services after their overall condition was stabilized. Patients with the history of recurrent stroke attacks and persistent deficits; with underlying psychotic and mental disorders; those who were handicapped before the stroke event; and the patients with comorbidities (e.g., heart failure, peritoneal or hemodialysis) that significantly affect the quality of life and limit life expectancy were excluded. Informed consent was received as much as possible from the patient but if there is aphasia, consent is obtained from the patient as well as the spouse or family member who is actively taking care of the patient and is defined as the proxy. The proxy then answered on behalf of the patient but in the presence of the patient.

## 4. Data Collection Instruments

Several validated well-developed tools were used in measurement of the changes: the stroke impact scale (SIS), the modified Rankin scale (mRS), Barthel index (BI), and the Beck depression iventory (BDI) [21–23]. The linguistic validated Hausa version of the stroke impact scale (SIS) version 3.0 which is a psychometrically robust 59-item stroke-specific self-reported measure was used to assess a number of physical, mental, and social dimensions of HRQoL [24]. Physical health was measured by four separate domains: strength, hand function, activities of daily living (ADL), and mobility; and these four domains were aggregated to create a composite “Physical domain.” Only the strength was rated in terms of “strength;” the other three physical dimensions were rated in terms of “amount of difficulty.” Mental health was measured by two domains: memory in terms of amount of difficulty and emotion in terms of frequency of events. The remaining two domains (social participation and communication) measured the participant's social health. The SIS 3.0 also assessed the patient's global perception of recovery, having the scores from 0 to 100 (where 0 score means no recovery and 100 meaning full recovery); hence the higher scores indicate the better HRQoL.

The modified Rankin Scale (mRS) assessed the degree of handicap as poststroke disability and measures the patient's ability to carry out daily living activities and determined whether they need any assistance. The Barthel index (BI) assessed the physical functional status and performance in daily living activities; (Barthel index score ranges from 0 (total dependence) to 100 (total independence)). Beck depression inventory (BDI), a-21-item self-reported screening tool, was used to assess the state of depression in the stroke survivors. All the four above-mentioned specific scales were used to measure the changes that occur in different aspects of health-related quality of life of Nigerian stroke survivors at 6 months and one year after stroke through a face to face interview with the patients and/or proxies. The socioeconomic, demographic, and clinical characteristics were collected using a questionnaire, also from medical and nursing records and from the treating doctors when needed.

## 5. Data Analysis

The data was analyzed using the Statistical Package for Social Science (SPSS) version 20.0. Descriptive statistics summarized the socioeconomic, demographic, and clinical characteristics of the participants and presented them as number with percentages; only the variable age was presented as mean with standard deviation. Paired samples *t*-test was used to calculate the amount of changes that occur in the physical, mental, and social health during the recovery phase. The forward stepwise linear regression model was used to identify the risk factors that predict the changes that occur in the physical, mental, and social dimension of health-related quality of life during recovery period. The *P* value of <0.05 is considered as the criterion for statistical significance.

In the regression model, the eight domains of stroke impact scale (SIS) and the composite physical domain were the dependent variables and the scores of the modified Rankin Scale, Barthel index, and Beck depression inventory were the independent variables. Socioeconomic and demographic variables such as age at the time of stroke, gender, religion, ethnicity, marital status, residency, education, occupation before and after stroke, income, who paid the hospital treatment expenditure, and status in the family and the clinical characteristics such as type of stroke, side of stroke, and caregiver during recovery phase were also the independent variables. In each regression analysis, the collinearity issue was also checked and only those predictors were included which are not highly correlated both at the 6 months and 1 year follow-up.

## 6. Results

Two hundred and thirty three stroke survivors participated in the study and they were surveyed at 6 months after stroke. Among them, 217 stroke survivors were followed up at 1 year.  Table 1 presents the socioeconomic, demographic, and clinical characteristics of 233 stroke survivors. The mean age (in years) of the respondents was 58.76 ± 13.24 and they were almost equally distributed by gender (male 51%). More than half (60%) of the respondents were married and nearly three quarter (74%) were urban residents. More than half (53%) of the respondents did not have enough income and in almost half (48%) of the cases, the family members paid the hospital treatment cost.

More than half of the respondents were diagnosed to suffer indeterminate type of stroke due to lack of proper diagnostic facilities. Among the diagnosed cases, ischemic stroke was the most common (37%). Slightly more than half (53%) of the respondents had right sided stroke. The majority of the stroke survivors were taken care of by their children (55%) and their spouses (22%).

The mean of different scales which measures the changes in physical and mental health during recovery phase of stroke (from 6 months to 1 year) is summarized in Table 2.

There are significant changes in all mean functional scores over the time period. The level of poststroke disability or dependence in performing daily life activities is significantly reduced which was measured by modified Rankin scale (mRS). Significant improvement in the activity daily living was also reflected in the BI scores. Both measurements indicate substantial improvement of physical health over the time period. Similar improvement was reflected in the score of BDI which suggests that the level of depression declined significantly from 6 months to 1 year after stroke.  Table 3 shows the changes that occurred in different domains of stroke impact scales from 6 months to 1 year. Except for the “emotion” domain, all different aspects of physical, memory, thinking process, and social health significantly improved during recovery period. The decreased emotion domain may be due to the fact that greater emphasis in stroke rehabilitation in Nigeria was on physical health by Physiotherapists. Stroke survivors do not have access to effective rehabilitation care such as by the occupational therapist to help them overcome the emotional consequences of stroke.

Table 4 presents the results of the forward stepwise linear regression models, both at 6 months and at 1 year. The adjusted *R* values of the composite physical domain were significant in the two time periods. The level of independency on functional status and depression, the ability to pay the hospital treatment cost, and who is the caregiver explained 69% of the variance on the outcome.

A change in 1 standard deviation of Barthel index (level of independency on functional status) has six times higher impact on physical health. The level of depression and dependency on others to pay the hospital treatment cost significantly affect the physical health status.

Ability to carry out daily living activities improved twice in patients who attended the rehabilitation centre as early as possible and when the caregiver is the spouse who could provide physiotherapy even at home within 6 months after stroke; and this ability is deteriorated when the stroke survivors have to depend on their children who are either not capable of taking care of them or cannot manage time to provide care to restore their functional life even after 1 year. Negative significant association exits between the level of depression and mental health, especially memory, thinking process, and emotion. Mental health was also affected by their economic condition and social disadvantage position such as a status of being a widow. The variables which measure the mental health in this study only can explain 29% of the variance of the outcome. There is significant association between the level of disability and functional status with social participation. Increased degree of disability reduced the social participation rate. Patients, who depend on social security to pay the hospital treatment cost and on children for care, are worse in communication.

## 7. Discussion

The study found significant improvement in functional disability among stroke survivors during recovery phase. Similar findings had been found in other studies [20, 25–28]. This has become common in many developed countries. like in USA, where the majority of stroke survivors receive rehabilitation care within 6 months after stroke to have the maximum possible level of independence [29]. A clinical trial research found that success of stroke rehabilitation depends on its early commencement, as soon as the survivor is medically stable and free from life threatening conditions [30]. It is already proven that stroke rehabilitation proved to be effective in improving functional outcomes and associated disability and mental depression [31–33]. Though this study could not identify how early the stroke survivors started to receive rehabilitation, it is realized that the Nigerian stroke survivors did obtain substantial benefits from poststroke rehabilitation which helped them to recover and to readjust with the changes. The study finding emphasizes the necessity for arranging early intervention to optimize the recoveries and rehabilitation for Nigerian stroke survivors.

Our study also found that involvement of the patients' spouse as the caregiver significantly improves the functional and mental status of the survivors. This finding corresponds with other study findings where the success of patient's self-management depends on the active partnership with key family member or caregivers [34, 35]. It is suggested that caregivers attend the rehabilitation sessions together with the survivors to actively manage and support the survivors at home. These sessions will allow the caregivers to remain informed about the patient's progress and to learn how to help the survivors at home to improve their physical, mental, and social functions.

Significant improvement in QOL was found except within the emotional domain. This is consistent with findings by [27, 36]. The stroke survivors could not overcome the emotional consequence of stroke even after 1 year which indicates the lack of emotional rehabilitation. This scenario was also described in developed countries decades ago [37]. Thus, the findings suggested including occupational therapists as direct-care providers, apart from physiotherapists, since they can place greater emphasis on objectifying the emotional consequences of stroke [38]. Occupational therapist can help them overcome the emotional consequences of stroke like frustration, dissatisfaction, and loss of confidence by giving them support to return to work [38]. Interdisciplinary partnership is recommended where all related health professionals including key specialists like physiotherapists and occupational therapists should collaborate as proactive health providers with key family members to ensure a comprehensive and early stroke rehabilitation program for effective poststroke recovery. This recommendation is in line with other research evidence [39] where interdisciplinary team approach was found effective in rehabilitation to help the disabled survivors to regain strength and range of movement, as well as to relearn the old skills or learn the new skills to carry out their daily living activities towards independent living.

Another finding is the relationship of emotional status to the lack of financial and social support. It is thus suggested to make the services freely available and accessible to all types of stroke survivors to avoid the emotional deterioration among the stroke survivors over time. Governments should develop effective health policy and strategy to promote early rehabilitation for stroke survivors to give them the better quality of life. Multidisciplinary specialist teams should be available at both inpatient and outpatient rehabilitation centers within hospitals and at the community. Generally, all doctors and nurses should receive specific training in poststroke rehabilitation. The role of occupational therapy should receive proper attention in the Nigeria health system. Community and home-based rehabilitation facilities and care should be available for those who are unable to move out of home. Effective public health interventions aimed to reduce the prevalence of modifiable risk factors and to prevent further stroke need to be designed. The study reinforces the need for governments of low income developing countries like Nigeria which are going through epidemiological transition to get ready in advance to tackle this emerging situation.

## 8. Conclusion

Stroke, due to its nature of sudden onset, usually leaves the individual and the family in an ill-prepared position to deal with its residual impairment in physical, mental, and social functions. Poststroke survival rate is anticipated to increase in developing countries due to better treatment and management; hence it generates the necessity for arranging rehabilitation for stroke survivors in order to give them the best possible quality of life. Key players must be involved in the interdisciplinary care together in partnership with family level caregiver to play the integral roles to improve physical and emotional health. Additional research on how religiosity, health care systems, and community factors influence the rehabilitation progress is warranted. Further research is also needed to identify those who are at risk of having early rehabilitation.

## Figures and Tables

**Table 1:** Socioeconomic, demographic, and clinical characteristics of the stroke survivors (*n* = 233).

Characteristics	Values
Age (years)	
Range	19 to 82
Mean	58.76 ± 13.24
Gender	
Male	118 (50.6%)
Female	115 (49.4%)
Marital status	
Single	5 (2.1%)
Married	139 (59.7%)
Widow/widower	70 (30%)
Divorced/separated	19 (8.2%)
Residence	
Urban	172 (73.8%)
Rural	61 (26.2%)
Income	
Enough and save	7 (3%)
Just enough	101 (43.3%)
Not enough	125 (53.6%)
Hospital treatment paid by	
Self-Support	63 (27%)
Friends	16 (6.9%)
Government support	1 (0.4%)
Social security	10 (4.3%)
Free	31 (13.3%)
Family support	112 (48.1)
Type of stroke	
Ischemic	86 (36.9%)
Hemorrhagic	24 (10.3%)
Indeterminate	123 (52.8%)
Side of stroke	
Right	123 (52.8%)
Left	110 (47.2%)
Caregiver during recovery phase	
Spouse	51 (21.9)
Children	128 (54.9)
In-law	2 (0.9)
Brother/sister/parents	49 (21)
Employer	3 (1.3)

**Table 2:** Changes in functional measures during recovery phase (6 months and one year after stroke).

Functional measure	Six months (*N* = 233) mean ± SD	One Year (*N* = 217) mean ± SD	Change mean ± SD
Modified Rankin scale (MRS)***	3.3 ± 1.1	3.1 ± 1.1	−0.2 ± 0.5
Barthel index (BI)***	60.5 ± 25.1	68.5 ± 18.8	8.0 ± 8.7
Beck depression inventory (BDI)***	12.3 ± 5.0	10.3 ± 4.0	−2.1 ± 1.9

Level of significance of *P* value: *P < 0.05; **P < 0.01; and ***P < 0.001.

**Table 3:** Changes in different dimensions of SIS during poststroke recovery phase.

Different dimensions of SIS	Six months (*n* = 233) mean ± SD	One year (*n* = 217) mean ± SD	Changes mean ± SD
Strength***	33.6 ± 21.5	37.6 ± 18.4	4.0 ± 7.0
Hand function***	28.7 ± 25.1	31.2 ± 25.3	2.5 ± 5.4
ADL***	46.4 ± 24.8	52.2 ± 22.7	5.4 ± 5.1
Mobility***	49.8 ± 30.5	54.1 ± 27.6	3.6 ± 4.7
Composite physical***	39.9 ± 21.3	43.8 ± 19.7	3.9 ± 3.2
Memory and thinking*	80.3 ± 22.2	80.9 ± 20.0	0.7 ± 4.1
Emotion***	57.4 ± 12.3	54.3 ± 12.6	−3.1 ± 10.0
Communication***	78.4 ± 24.5	79.6 ± 21.8	1.2 ± 4.9
Participation**	39.8 ± 23.6	41.0 ± 22.4	1.2 ± 5.8
Global recovery***	57.2 ± 19.9	62.9 ± 15.2	5.7 ± 7.5

Level of significance of *P* value: *P < 0.05; **P < 0.01; and ***P < 0.001.

**Table 4:** Predictors of stroke impact scale domains during poststroke recovery phase.

Stroke impact scale domains	Six months Follow-up			One year Follow-up		
	Standardized coefficient	Standard error	*R* ^2^ adjusted	Standardized coefficient	Standard error	*R* ^2^ adjusted
Strength			**0.51**			**0.50**
Modified Rankin scale	−0.523***	1.067		−0.337***	1.424	
Beck depression inventory score	−0.265***	0.238		−0.149*	0.287	
Hand function			**0.33**			**0.36**
Barthel index	0.351***	0.061		0.402***	0.083	
Age	0.220**	0.123		0.285***	0.119	
Caregiver (spouse versus employer)	0.240**	4.453		0.236**	4.309	
Activities of daily living (ADL)			**0.64**			**0.63**
Barthel index	0.722***	0.040		0.625***	0.055	
Caregiver (spouse versus employer)	0.206***	2.599				
Caregiver (children versus employer)				−0.182***	2.034	
Hospital treatment charge (Self-support versus family support)	0.166***	2.259		0.132**	2.221	
Hospital treatment charge (social security versus family support)	−0.143**	5.001		−0.192***	4.615	
Mobility			**0.70**			**0.65**
Barthel index	0.825***	0.044		0.767***	0.060	
Income (enough and save versus not enough)	0.123**	6.369				
Hospital treatment charge (self-support versus family support)				0.143**	2.603	
Composite physical domain			**0.69**			**0.69**
Barthel index	0.650***	0.042		0.567***	0.069	
Caregiver (children versus employer)	−0.195***	1.768		−0.217***	1.657	
Hospital treatment charge (Social security versus family support)	−0.147***	3.915		−0.127**	3.743	
Beck depression inventory score	−0.184***	0.208				
Memory and thinking			**0.27**			**0.29**
Beck depression inventory score	−0.246***	0.284		−0.236***	0.324	
Caregiver (Spouse versus employer)	0.341***	3.485		0.183*	3.632	
Age	0.230**	0.115		0.179*	0.106	
Emotion			**0.29**			**0.29**
Beck depression inventory score	−0.317***	0.178		−0.217**	0.194	
Marital status (widow versus divorce)	−0.229***	1.592		−0.193**	1.672	
Hospital treatment charge (Self-support versus family support)	0.192**	1.651		0.227***	1.759	
Income (Just enough versus not enough)				0.220***	1.495	
Communication			**0.41**			**0.40**
Caregiver (children versus employer)	−0.271***	2.487		−0.230***	2.712	
Hospital treatment charge (social security versus family support)	−0.180**	5.709		−0.157**	6.430	
Social participation			**0.56**			**0.56**
Age	0.264***	0.088		0.173**	0.092	
Barthel index	0.382***	0.082		0.350***	0.096	
Modified Rankin scale	−0.305***	1.657		−0.234**	1.791	
Hospital treatment charge (Self-support versus family support)	0.245***	2.507		0.159**	2.492	

Level of significance of *P* value: *P < 0.05; **P < 0.01; and ***P < 0.001.
